# A Rapid Method for Obtaining the Transgenic Roots of Crassulaceae Plants

**DOI:** 10.3390/plants13213024

**Published:** 2024-10-29

**Authors:** Lan Zhou, Yulu Yang, Anket Sharma, Vijay Pratap Singh, Durgesh Kumar Tripathi, Wona Ding, Muhammad Junaid Rao, Bingsong Zheng, Xiaofei Wang

**Affiliations:** 1 State Key Laboratory of Subtropical Silviculture, Zhejiang A&F University, Hangzhou 311300, China; 2 Plant Physiology Laboratory, Department of Botany, C.M.P. Degree College, A Constituent Post Graduate College of University of Allahabad, Prayagraj 211002, Uttar Pradesh, India; 3 Crop Nanobiology and Molecular Biology Laboratory, Amity Institute of Organic Agriculture (AIOA), Amity University, Noida 201303, Uttar Pradesh, India; 4 College of Science and Technology, Ningbo University, Ningbo 315300, China

**Keywords:** Crassulaceae, *Agrobacterium*-mediated transformation, *GFP* reporter, *RUBY* reporter, transformation system

## Abstract

Crassulaceae plants are valued for their horticultural, ecological, and economic significance, but their genetic improvement is hindered by the absence of efficient and stable genetic transformation methods. Therefore, the development of a tailored genetic transformation method is crucial for enhancing the progress of the genetic improvement of Crassulaceae plants. The results indicate that, in the transformation experiments conducted on *Kalanchoe tetraphylla*, the K599 strain exhibited the highest transformation efficiency (76.67%), while C58C1 was least efficient (21.43%). An acetosyringone concentration of 100 μM was optimal for the hairy root transformation, and the immersion method yielded the highest efficiency. Additionally, the Silwet L-77 concentration significantly influenced the transformation efficiency, with 0.05% leading to a decrease. Upon four Crassulaceae species, notable differences were observed, with *K. tetraphylla* exhibiting the highest efficiency of 100%, and *Sedum alfredii* displaying the lowest efficiency of 5%. The *RUBY* reporter gene offers a more distinct advantage over *GFP* in observing the transformation effects. This study developed a simple, feasible, and cost-effective method for obtaining transgenic roots from leaves of Crassulaceae. The methodology provides technical support for the genetic improvement and gene function research of Crassulaceae plants.

## 1. Introduction

Crassulaceae plants hold significant importance across various domains, including horticulture, ecology, and economics. In horticulture, these plants are highly valued for their aesthetic appeal, with many species being cultivated for ornamental purposes due to their unique foliage, vibrant flowers, and interesting growth habits [1]. Ecologically, Crassulaceae plants play a crucial role in maintaining biodiversity and ecosystem function, often serving as key components in arid and semi-arid ecosystems, where they are well adapted to survive extreme conditions [2]. Their ability to perform Crassulacean acid metabolism (CAM) photosynthesis allows them to thrive in environments with limited water, thereby contributing to ecosystem stability and resilience [3,4]. Economically, Crassulaceae plants have significant contributions to landscaping, floral arrangements, and the pharmaceutical industry [5,6,7]. The global demand for Crassulaceae plants has surged due to their low maintenance and drought resistance, making them popular in landscaping and interior design [8]. In addition, some species within the family possess medicinal properties, being used in traditional medicine for their anti-inflammatory, antimicrobial, and wound-healing effects [9]. These multifaceted uses highlight the broad significance of Crassulaceae plants and underscore the importance of advancing their genetic improvement for further utilization in these areas.

Despite their importance, Crassulaceae plants face significant challenges in genetic improvement, particularly in breeding, gene function exploration, and enhancing stress resistance. Traditional breeding methods are constrained in terms of the speed and scope of genetic changes they can achieve. However, with the completion of genome sequencing for an increasing number of Crassulaceae species [10,11,12,13,14], the potential for targeted genetic improvement has increased dramatically. Genomic data allow researchers to better understand the genetic architecture of these plants, enabling the identification and manipulation of genes associated with desirable traits, such as increased drought tolerance, adaptability to climate change or heavy metal accumulation, which has notable applications in bioremediation [8,15,16,17,18]. Nonetheless, the effective utilization of this genetic knowledge hinges on the development of efficient genetic transformation technologies.

Currently, several Crassulaceae species have successfully established *Agrobacterium*-mediated genetic transformation systems under tissue culture conditions. For example, *Kalanchoe blossfeldiana* has been transformed, using both *A. tumefaciens* and *A. rhizogenes* [19], whereas *Sedum plumbizincicola* is exclusively transformed using *A. tumefaciens* [20]. However, tissue-culture-based transformation methods come with various challenges, such as the need for precise explant sterilization, labor-intensive callus induction processes, difficulties in preventing bacterial contamination, and the high cost and time demands of training skilled operators [21]. Additionally, *A. tumefaciens* exhibits substantial species-dependent transformation efficiencies. For instance, Cho et al. (2011) screened 30 different Crassulaceae species and ultimately selected *K. pinnata* for developing a transformation system, highlighting the limitations in species versatility [22].

In contrast, transformation via *A. rhizogenes* shows reduced species dependency [23]. Studies by Collier et al. (2005) demonstrated high transformation efficiencies across 14 species from different plant families and genera [24]. Similarly, Meng et al. (2021) observed high transformation rates in nine species of both herbaceous and woody plants, indicating that *A. rhizogenes* can effectively transform a broader range of species [25]. This reduced species dependency suggests that *A. rhizogenes* is a promising tool for Crassulaceae transformation.

Given that many Crassulaceae species readily propagate via leaf cuttings [26], this study focused on establishing a genetic transformation system using leaf explants under non-tissue culture conditions. The system involves screening different *A. rhizogenes* strains, testing various infection methods, and optimizing acetosyringone (As) concentrations. This streamlined system was then applied across several Crassulaceae species, offering a versatile and practical approach to support gene function research and future genetic improvements in these plants.

## 2. Results

### 2.1. Effects of Different Infection Methods on Transformation Efficiency

**Figure 1:**
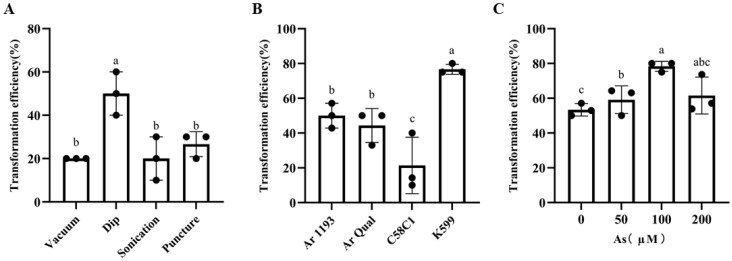
Selection of the optimal conditions for infection. (**A**) The transformation efficiency of different infection methods (dip, stab, vacuum, and ultrasound) were tested; (**B**) The transformation efficiency of four *A. rhizogenes* strains (K599, C58C1, Ar Qual, and Ar 1193) were tested; (**C**) The transformation efficiency of the different As concentrations (0, 50, 100, 200 μM) were tested. The data presented were the mean ± standard error of three independent replicate experiments. Different lower case letters indicated a significant difference between the explants (*p* < 0.05).

Infection methods significantly influence the transformation efficiency of plants. We tested four infection methods (dip, vacuum, sonication, and puncture) on *Hylitalephium spectabile*. The dip method achieved the highest transformation efficiency, with a positive rate of 50%. In contrast, the vacuum, sonication, and puncture methods had lower efficiencies, with each around 20% (Figure 1A). The lower survival rate of leaves in the vacuum, sonication, and puncture methods may be due to tissue damage caused by these techniques, reducing the plants’ ability to recover. We selected the dip method for the following transformation.

### 2.2. Effect of Agrobacterium rhizogenes Strains

Transformation efficiency can vary among different *A. rhizogenes* strains depending on the plant species. To investigate this, we performed transformation experiments on *H. spectabile* using four *A. rhizogenes* strains (K599, C58C1, Ar Qual, and Ar 1193) by the dip infection method. The results showed that the K599 strain exhibited the highest transformation efficiency, reaching 76.67%, while the C58C1 strain displayed the lowest efficiency at 21.43% (Figure 1B). Based on these findings, we used the K599 strain in subsequent experiments.

### 2.3. Effect of As Concentration on the Transformation Efficiency

To optimize hairy root transformation in Crassulaceae plants, different concentrations of As were tested to assess their impact on transformation efficiency, using the K599 strain with the dip infection method. The results indicated that, in the absence of As, the transformation efficiency was 53.35%. The transformation efficiency reached 78.33% with an As concentration of 100 μM. However, beyond this concentration, the efficiency declined (Figure 1C). Therefore, we chose 100 μM of As in the following experiments.

### 2.4. Effect of Silwet L-77 on the Transformation Efficiency

**Figure 2:**
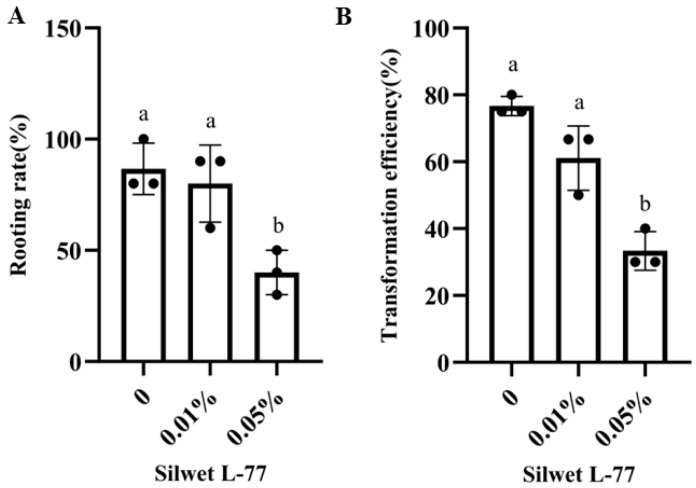
Effects of different concentrations of Silwet L-77 on rooting rate and transformation efficiency. (**A**) The rooting rate of Silwet L-77 (0, 0.01%, 0.05%); (**B**) The transformation efficiency of Silwet L-77 (0, 0.01%, 0.05%). Data presented were the mean ± standard error of three independent replicate experiments. Different lower case letters indicate a significant difference between the explants (*p* < 0.05).

Silwet L-77, a surfactant, is known to reduce surface tension, facilitating the penetration of infection solutions into leaf tissues and potentially improving the transformation efficiency [27]. Under the optimal transformation conditions, we tested two concentrations of Silwet L-77, 0.01% and 0.05%, on *H. spectabile* to assess their effects. The results showed no significant difference in transformation efficiency between 0.01% Silwet L-77 and the control (without Silwet L-77). However, as the concentration increased to 0.05%, the transformation efficiency decreased (Figure 2A). Additionally, the rooting rate of leaves declined with an increasing Silwet L-77 concentration (Figure 2B), likely due to tissue necrosis induced by higher surfactant levels.

### 2.5. Optimized Transformation Protocol

**Figure 3:**
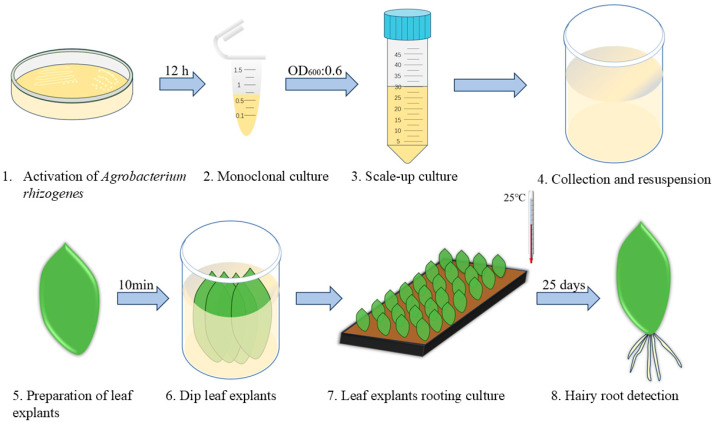
Workflow for *A. rhizogenes*-mediated transformation of Crassulaceae plants.

Based on our experiments, we identified the optimal conditions for genetic transformation of *H. spectabile* under non-tissue culture conditions. These conditions included using the K599 strain, adjusting the bacterial culture to an OD_600_ of 0.6, adding 100 µM As to the suspension, and employing the dip infection method. Cultivation in a growth chamber (12 h light at 25 °C and 12 h dark at 18 °C) for 25–30 days results in the successful generation of transgenic hairy roots (Figure 3). Compared to the use of traditional tissue culture methods, this approach is faster and more cost-effective.

### 2.6. Differences in Transformation Efficiency Among Crassulaceae Species

**Figure 4:**
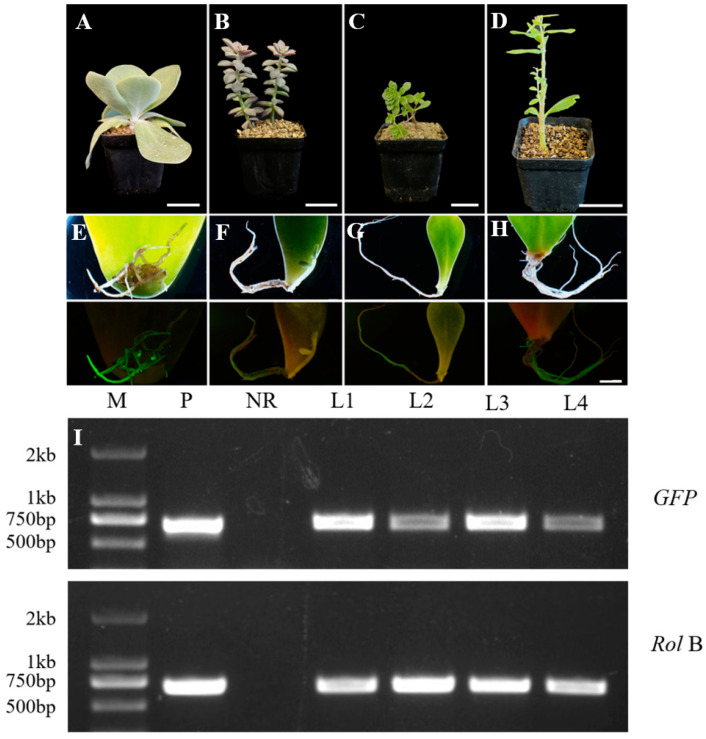
Four Crassulaceae species transformation, GFP reporter observation, and PCR analysis. Four Crassulaceae species used for transformation, from left to right, were *K. tetraphylla* (**A**), *G. paraguayense* (**B**), *S. alfredii* (**C**), and *H. spectabile* (**D**), with a scale bar of 5 cm. Transgenic hairy roots from leaf explants of *K. tetraphylla* (**E**), *G. paraguayense* (**F**), *S. alfredii* (**G**), and *H. spectabile* (**H**), the top and bottom images in (**E**–**G**) were captured using bright filed and fluorescence microscopy, respectively, with a scale bar of 5 mm. (**I**) Validation of transgenic hairy roots using PCR analysis. M: 2000 marker; P: positive control; N: negative control; L1–L4: hairy roots from *K. tetraphylla*, *G. paraguayense*, *S. alfredii*, and *H. spectabile*.

To assess the feasibility and efficiency of the developed genetic transformation method for Crassulaceae plants, we compared the transformation efficiency across four Crassulaceae species: *Graptopetalum paraguayense*, *Hylotelephium spectabile*, *Kalanchoe tetraphylla*, and *Sedum alfredii*. The positive hairy roots were observed in all species tested (Figure 4A–H) and confirmed by PCR analysis (Figure 4I). The transformation efficiency varied significantly. *K. tetraphylla* had the highest efficiency, reaching 100%, whereas *S. alfredii* had the lowest, with only 5.00% (Table 1). We also found that there was little difference in the rate of rooting between four Crassulaceae species (Table 1). These findings suggest that the transformation efficiency is species-dependent in Crassulaceae plants, and not related to rooting efficiency.

### 2.7. Application of the RUBY Reporter in Crassulaceae Plant Transformation

**Figure 5:**
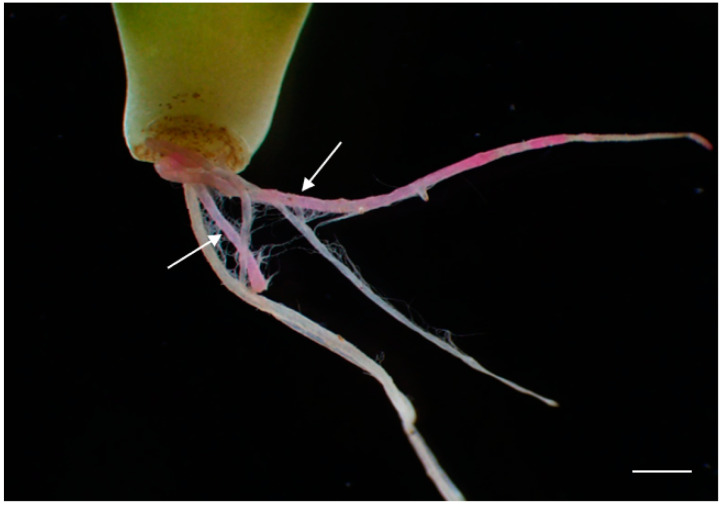
Representative photographs of the *RUBY* reporter applied to Crassulaceae plant transformation. The white arrows represent the hairy roots expressing the *RUBY* gene. Bar = 2 mm.

Reporter genes are widely used to visualize gene expression, and the *RUBY* gene offers distinct advantages over *GFP* [28]. RUBY converts tyrosine into bright red betacyanins, allowing gene expression to be observed directly without special equipment or chemical treatments. Using the *35S::RUBY* vector, we successfully transformed *H. spectabile* and observed the growth of red-positive roots (Figure 5). While the color was barely visible in the callus or early stages of root development, the red pigmentation became more pronounced as the root matured, especially in the mature root zone. The red pigmentation also appeared with different intensities on different roots. This experiment demonstrated that the *RUBY* reporter gene was a suitable reporter for Crassulaceae plants.

## 3. Discussion

Crassulaceae plants occupy a unique position in both horticulture and ecology. Despite their environmental and economic potential, the genetic improvement of Crassulaceae plants faces significant challenges. One major obstacle is the lack of efficient and reliable genetic transformation methods. Traditional approaches to genetic modification primarily rely on the use of *A. tumefaciens* under tissue culture conditions, which are labor-intensive, time-consuming, and often yield low transformation efficiencies. These limitations hinder the development of new varieties with improved traits. This study directly addresses these challenges by developing a novel genetic transformation method for Crassulaceae plants using *A. rhizogenes*. This method overcomes many of the shortcomings of traditional approaches, providing a simpler, faster, and more efficient route for genetic transformation in Crassulaceae species. By optimizing key factors such as bacterial strain selection, infection methods, and transformation conditions, we have established an efficient system.

During the genetic transformation process, selecting the appropriate *A. rhizogenes* strain is crucial as it significantly influences the success rate and efficiency of the transformation [29]. In this study, we tested four *A. rhizogenes* strains (K599, C58C1, Ar Qual, and Ar 1193) for their ability to induce hairy root formation in Crassulaceae plants, particularly *Kalanchoe*. Among the strains tested, K599 consistently outperformed the others, achieving a transformation efficiency of 76.67%, while C58C1 demonstrated the lowest efficiency at 21.43%, suggesting that K599 is more suitable for Crassulaceae plant transformation. The high efficiency of K599 can be attributed to its ability to effectively facilitate T-DNA transfer from bacteria to plant cells, crucial for successful transformation and hairy root induction. These findings align with previous studies identifying K599 as a highly efficient strain for inducing hairy roots [25,29]. Another research on *S. alfredii* also found K599 to be the most effective strain for transformation, further validating its applicability in Crassulaceae plants [30].

Another critical factor affecting the efficiency of *A. rhizogenes*-mediated transformation in Crassulaceae plants is the concentration of As. As a phenolic compound, As plays a pivotal role in activating the virulence genes of *Agrobacterium*, thereby enhancing its ability to transfer T-DNA into plant cells [31,32]. Concentrations of As are typically in the range of 20–200 µM, depending on the plant species [31,32,33,34]. In this study, various concentrations of As were tested to determine the optimal conditions for hairy root transformation, with 100 μM As emerging as the most effective concentration. At this concentration, the highest transformation efficiency of 78.33% was achieved, striking a balance between sufficient virulence gene activation and minimal toxicity to plant tissues. Notably, without As, the transformation efficiency also reached 53.35%. *A. rhizogenes* is known to act as a ‘natural genetic engineer’. In nature, in the absence of As, wild *A. rhizogenes* can induce characteristic roots at the site of infection, resulting from the introduction of bacterial genetic material (T-DNA) into the plant genome [35,36].

Beyond optimizing the As concentration, the choice of infection method is crucial for achieving high transformation efficiency. In this study, four different infection techniques were evaluated: dip, vacuum, sonication, and puncture infection [37,38]. The dip method outperformed the others, yielding the highest transformation efficiency of 50%, compared to 20% for other methods. The dip method’s superiority can be attributed to its simplicity and gentleness on plant tissues. By dipping plant leaves in a bacterial suspension, this method ensures direct contact between numerous *Agrobacterium* cells and plant tissues, enhancing the infection efficiency. Furthermore, the dip method minimizes physical damage to plant tissues, crucial for maintaining explant viability during the transformation process. In contrast, the vacuum, sonication, and puncture infection involve the more aggressive manipulation of plant tissues, potentially increasing tissue damage and reducing the transformation efficiency.

Silwet L-77, a non-ionic surfactant, is widely used in plant transformation studies to reduce liquid surface tension, facilitating the penetration of a bacterial suspension into plant tissues [39]. The use of Silwet L-77 and centrifuge pre-treatment has already been shown to increase the efficiency of transformation in a number of plant species [40,41]. However, in this study, Silwet L-77’s role in *A. rhizogenes*-mediated Crassulaceae plant transformation yielded mixed results. At a concentration of 0.01%, Silwet L-77 had minimal impact on transformation efficiency, while at 0.05%, a significant reduction in efficiency was observed. This suggests that, while Silwet L-77 can enhance the bacterial cell–plant tissue interactions, its concentration must be carefully controlled to avoid adverse effects on the transformation process. At higher concentrations, Silwet L-77 may disrupt cell membranes, leading to tissue necrosis and reduced transformation efficiency [42].

Another key finding of this study was the significant variation in transformation efficiencies among the four Crassulaceae species tested: *K. tetraphylla*, *S. alfredii*, *G. paraguayense*, and *H. spectabile*. *K. tetraphylla* exhibited a remarkable transformation efficiency of 100%, while *S. alfredii* showed only 5%, indicating differential sensitivity to *A. rhizogenes*-mediated transformation among species. Understanding these variations can provide insights into the biological or genetic factors influencing the transformation success in Crassulaceae plants. *K. tetraphylla’*s high efficiency may be attributed to its cell wall structure, genetic background, and compatibility with *A. rhizogenes*. In contrast, *S. alfredii’*s lower efficiency may stem from a more complex cell wall structure or robust defense mechanism [16].

Reporter genes play a vital role in tracking gene expression and transformation success in genetic transformation studies [43]. Traditionally, *GFP* has been the standard report gene due to its ability to fluoresce under specific wavelengths, enabling the visual monitoring of gene activity. However, this study highlights the advantages of the *RUBY* reporter gene as an alternative to *GFP*, particularly in terms of practicality and ease of detection. RUBY produces visible red betalains, allowing for the naked-eye detection of transgenic tissues, simplifying the gene expression-monitoring process [28,44]. In this study, we successfully introduced RUBY into Crassulaceae plants and made it easy to observe the successful transformation event.

The rapid *A. rhizogenes*-mediated transformation method developed in this study offers significant practical advantages, particularly in addressing root-related gene function analysis for Crassulaceae plants. During our experiment, another research group developed a similar method for succulents and obtained regenerated plants [45]. The study investigates a modified cut-dip-budding (CDB) method, successfully transforming three succulent species: *K. blossfeldiana*, *Crassula arborescens*, and *Sansevieria trifasciata*. *K. blossfeldiana* achieved the highest transformation efficiency (74%), followed by *C. arborescens* (5%) and *S. trifasciata* (3.9–7.8%). These results underscore the variability in transformation efficiency across different succulent species, with monocotyledonous species like *S. trifasciata* being more challenging to transform than dicotyledonous species. Similarly, in our study, *K. tetraphylla* demonstrated a notably high transformation efficiency (76.67%) using *A. rhizogenes* K599, while other species, such as *S. alfredii*, showed much lower efficiencies (5%). This significant species-dependent variability highlights the need to tailor transformation methods to specific plants within a genus or species. Understanding these differences also offers a promising direction for future research into the underlying mechanisms driving transformation efficiency. Furthermore, many Crassulaceae species possess the ability to regenerate shoots from cut leaves, which suggests the potential for regenerating whole transgenic plants in future studies.

## 4. Materials and Methods

### 4.1. Plant Material and Growth Condition

In this study, four species of succulent plants were selected for experimentation: *G. paraguayense*, *H. spectabile*, *K. tetraphylla*, and *S. alfredii*. Healthy, intact, and undamaged leaves were collected from these plants. All specimens were grown in a controlled growth chamber under the following conditions: 25 °C, 50% humidity, and a 12 h light/dark photoperiod.

### 4.2. Agrobacterium rhizogenes and Vectors

*A. rhizogenes* strains K599, C58C1, Ar Qual, and Ar 1193 were used as experimental strains to induce hairy root formation in *H. spectabile* and to compare their transformation efficiencies. The binary vector pCAMBIA1300 containing the *35S::eGFP* or *35S::RUBY* construct was introduced into *A. rhizogenes* using the freeze–thaw method. The procedure was as follows: *A. rhizogenes* competent cells stored at −80 °C were allowed to partially thaw at room temperature, then placed on ice when in a slushy state. A 1 μL plasmid was added to the competent cells, and the tube was quickly tapped to mix the solution. The mixture was incubated sequentially: 5 min on ice, 5 min in liquid nitrogen, 5 min in a 37 °C water bath, and 5 min on ice again. Afterward, 500 μL of LB liquid medium without antibiotics was added, and the mixture was shaken at 28 °C for 2–3 h. Approximately 50 μL of the bacterial culture was then spread on LB agar plates containing 50 mg dm^−3^ kanamycin (kan) and 50 mg dm^−3^ streptomycin (str), which were inverted and incubated in a 28 °C incubator for 2 days.

### 4.3. Agrobacterium rhizogenes-Mediated Transformation

*A. rhizogenes* was inoculated onto LB solid medium containing 50 mg dm^−3^ str and 50 mg dm^−3^ kan, and incubated at 28 °C for 2 days. A single colony was picked and shaken in liquid LB medium for 12 h, followed by expansion until the OD_600_ value reached 0.6. The bacterial culture was centrifuged at 5000 rpm for 10 min, and the supernatant was discarded. The bacterial pellet was resuspended in a solution containing 2.13 g dm^−3^ MES, 2.03 g dm^−3^ MgCl_2_·6H_2_O with the pH adjusted to 5.2 using KOH, and supplemented with As. Leaves from Crassulaceae plants were submerged in the resuspension for 10 min, then transferred to the moistened vermiculite (3–6 mm particle size). The leaves were incubated in the dark at 25 °C for 2 days, followed by a 12 h light/12 h dark cycle in a growth chamber (25 °C light, 18 °C dark) for 25–30 days before data collection.

To optimize the transformation procedure, we initially used the *A. rhizogenes* strain K599 at an OD_600_ of 0.6 and an As concentration of 100 µM. Four distinct infection methods were tested with the bacterial suspension: (1) Vacuum infiltration: Leaves were immersed in the bacterial suspension, vacuum infiltrated at 0.08 Mpa for 5 min, followed by an additional 5 min of immersion. (2) Dip method: Leaves were simply immersed in the bacterial suspension for 10 min, without any additional physical treatment. (3) Sonication: Leaves were immersed in the bacterial suspension, subjected to sonication at 60 Hz for 1 min, followed by an additional 9 min of immersion. (4) Puncture method: Leaves were soaked in the bacterial suspension and then punctured multiple times with a sterile needle to facilitate bacterial penetration. After selecting the dip method based on its performance, we optimized the procedure further using four *A. rhizogenes* strains (K599, C58C1, Ar Qual, and Ar 1193), all at an OD_600_ of 0.6 and As at 100 µM. To evaluate the effect of different As concentrations, we tested four concentrations (0, 50, 100, and 200 µM) using the K599 strain (OD_600_ of 0.6) with the dip method. Additionally, we tested the effect of two Silwet L-77 concentrations (0.01% and 0.05%) by adding Silwet L-77 to the immersion medium, using the K599 strain (OD_600_ of 0.6) and As at 100 µM, with the dip method.

### 4.4. Hairy Root Observation

To assess the presence of hairy roots, leaves were removed from the vermiculite, and a handheld fluorimeter (LUYOR-3410RB) was used to rapidly screen for positive roots. In this study, positive roots refer to roots that exhibit green fluorescence, confirming successful transgene integration. If positive roots were detected, the vermiculite was carefully removed, and surface moisture was blotted with paper. Roots were then photographed under a stereo fluorescence microscope (Olympus, Tokyo, Japan).

### 4.5. DNA Extraction and PCR Analysis

Hairy root DNA was extracted using the Plant DNA Quick Extraction Kit (Zenbio, Hangzhou, China). The protocol was as follows: hairy roots (1–2 cm in length) were excised and placed in a 2 mL centrifuge tube, then ground in liquid nitrogen. After grinding, 200 μL of Buffer A was added, and the sample was vortexed thoroughly. Next, 200 μL of Buffer B was added, followed by centrifugation at 12,000× *g* for 1 min. The supernatant was diluted 20-fold for PCR analysis. The *eGFP* fragment was amplified using the primers eGFP-F (5′-ATGGTGAGCAAGGGCGAGGAGCTGTTCACC-3′) and eGFP-R (5′-TTACTTGTACAGCTCGTCCATGCCGTGAGTGATCC-3′). Additionally, the Rol B gene fragment was amplified using the primers Rol B-F (5′-GCCAGCATTTTTGGTGAACT-3′) and Rol B-R (5′-GGCACTGAACTTGCCGTTAT-3′).

### 4.6. Data Analysis

Statistical analysis was performed using SPSS 25, with analysis of variance (ANOVA) used to assess the significant differences in the data. Graphs were generated using GraphPad Prism 10. Differences were considered significant at the *p* < 0.05 level, with significant results indicated by different letters.

## 5. Conclusions

In summary, we developed a rapid and highly efficient genetic transformation system specifically tailored to Crassulaceae plants that effectively overcome the limitations of conventional transformation methods. This innovative approach, using *A. rhizogenes* and leaf explants, operates under non-tissue culture conditions and achieves transgenic root generation within 25–30 days. This streamlined method not only simplifies material sourcing, but also eliminates the need for specialized technical skills, allowing genetic transformation to be performed with basic equipment. As such, it represents a promising technique for unravelling gene function in Crassulaceae, significantly advancing our understanding of these versatile plants.

## Figures and Tables

**Table 1:** Rooting rate and positive rooting rate of four Crassulaceae species.

Genotypes	Rooting Rate (%)	Positive Rooting Rate (%)
*Kalanchoe tetraphylla*	96.11	100
*Graptopetalun paraguayense*	98.33	29.24
*Sedum alfredii*	86.67	5.00
*Hylitalephium spectabile*	86.67	70.42

## Data Availability

Datasets generated for this study are available upon request from the corresponding author.
